# On the paternal origin of trisomy 21 Down syndrome

**DOI:** 10.1186/1755-8166-3-4

**Published:** 2010-02-23

**Authors:** Maj A Hultén, Suketu D Patel, Magnus Westgren, Nikos Papadogiannakis, Anna Maria Jonsson, Jon Jonasson, Erik Iwarsson

**Affiliations:** 1 Warwick Medical School, University of Warwick, UK; 2 Department of Biological Sciences, University of Warwick, UK; 3 Department of Obstetrics and Gynecology, Karolinska Institutet, Sweden; 4 Department of Pathology, Karolinska Institutet, Sweden; 5 Department of Clinical and Experimental Medicine, Linköping University, Sweden; 6 Department of Molecular Medicine and Surgery, Karolinska Institutet, Sweden

## Abstract

**Background:**

Down syndrome (DS), characterized by an extra free chromosome 21 is the most common genetic cause for congenital malformations and learning disability. It is well known that the extra chromosome 21 originates from the mother in more than 90% of cases, the incidence increases with maternal age and there is a high recurrence in young women. In a previous report we have presented data to indicate that maternal trisomy 21 (T21) ovarian mosaicism might provide the major causative factor underlying these patterns of DS inheritance. One important outstanding question concerns the reason why the extra chromosome 21 in DS rarely originates from the father, i.e. in less than 10% of T21 DS cases. We here report data indicating that one reason for this parental sex difference is a very much lower degree of fetal testicular in comparison to ovarian T21 mosaicism.

**Results:**

We used fluorescence in situ hybridisation (FISH) with two chromosome 21-specific probes to determine the copy number of chromosome 21 in fetal testicular cell nuclei from four male fetuses, following termination of pregnancy for a non-medical/social reason at gestational age 14-19 weeks. The cells studied were selected on the basis of their morphology alone, pending immunological specification of the relevant cell types. We could not detect any indication of testicular T21 mosaicism in any of these four male fetuses, when analysing at least 2000 cells per case (range 2038-3971, total 11.842). This result is highly statistically significant (p < 0.001) in comparison to the average of 0.54% ovarian T21 mosaicism (range 0.20-0.88%) that we identified in eight female fetuses analysing a total of 12.634 cells, as documented in a previous report in this journal.

**Conclusion:**

Based on these observations we suggest that there is a significant sex difference in degrees of fetal germ line T21 mosaicism. Thus, it would appear that most female fetuses are T21 ovarian mosaics, while in sharp contrast most male fetuses may be either very low grade T21 testicular mosaics or they may be non-mosaics. We further propose that this sex difference in germ line T21 mosaicism may explain the much less frequent paternal origin of T21 DS than maternal. The mechanisms underlying the DS cases, where the extra chromosome 21 does originate from the father, remains unknown and further studies in this respect are required.

## Background

It is now just about 50 years since the genetic background for Down syndrome (DS) was identified [1-3] with the most common reason being an extra free chromosome 21, trisomy 21 (T21). Long before then Penrose (as well as some other authors) had suggested that the condition could be caused by a chromosome abnormality; and at the same time he documented a strong maternal age effect with an increasing incidence of DS births to mothers at later reproductive ages [4,5]. Remarkably, a couple of years before the confirmation of the true chromosomal background he also identified a biomarker for germ line and somatic chromosomal mosaicism (the typical dermatopglyphics) in parents and sibs [6]. In the interim it has become clear, primarily by family linkage studies tracing DNA markers along the length of chromosome 21q between parents and children in DS families that the majority of T21 DS cases inherit the extra chromosome 21 from their mother (more than 90%) while in only a minority (less than 10%) the extra chromosome 21 originates from the father [7-11].

Importantly, the underlying mechanism for this parental sex difference still remains unknown. Nevertheless, it has been generally accepted that the main problem is mal-segregation of chromosomes 21 in an original disomy 21 oocyte, a dogma most recently re-iterated by Oliver et al. 2008, 2009, Cheng et al. 2009, Fledel-Alon et al. 2009 and Cheung et al. 2010 [10,12-15]. Thus it is thought that the segregation of chromosomes 21, taking place at ovulation and after fertilisation in women post puberty is particularly vulnerable and prone to non-disjunction dependent on abnormalities in chiasma formation leading to mechanical instability. It is also generally accepted that a number of other genetic and environmental factors may contribute to the variation in chance of having a child with T21 DS (see e.g. Hunt et al. 2008, Jones 2008, Oliver et al. 2008, Allen et al. 2009, Coppedè 2009, Driscoll et al. 2009, Garcia-Cruz et al. 2009, Ghosh et al. 2009, Hassold and Hunt 2009, Keefe and Liu 2009, Mailhes 2008, Martin 2008, Migliore et al. 2009, Vogt et al. 2009 [8-10,16-26]).

We have recently challenged this dogma by suggesting that the most likely predisposing factor in women for T21 conceptions is instead the common occurrence of fetal ovarian T21 mosaicism and in particular the net result of the behaviour of any such T21 oocytes during development from fetal life until adulthood and maturation for ovulation [27,28]. Based on the observation that all the eight fetuses investigated in this respect, where termination of pregnancy had been performed for a non-medical/social reason, showed ovarian mosaicism with an average of 0.54% T21 cells (range 0.20-0.88%, SD 0.23) we concluded that most females might be low grade T21 mosaics. On the other hand, some exceptional women, who are high grade T21 mosaics, will be predisposed to T21 conceptions already at an early reproductive age and endure an associated high recurrence risk [25,29].

We have here explored the possibility that the low incidence of T21 of paternal origin is correlated to a lower or insignificant level of germinal/gonadal mosaicism in men in comparison to women. Our data are consistent with this hypothesis. Using fluorescence in situ hybridisation (FISH) with dual chromosome 21-specific probes, we have not found a single T21 cell nucleus in a sample of nearly 12.000 relevant cell nuclei from fetal testes, obtained from four male fetuses where termination of pregnancy had been obtained for a non-medical/social reason. These data are highly statistically different from those obtained in our previous study of more than 12.000 cells by screening eight fetal ovaries, where on average we identified one cell in 200 with T21 without any statistically significant inter-individual variation. We further find it highly unlikely that, akin to the situation in women [27] any rare fetal testicular T21 cells that have remained undetected in our study would accumulate during spermatogenesis post puberty.

## Results and Discussion

**Figure 1:**
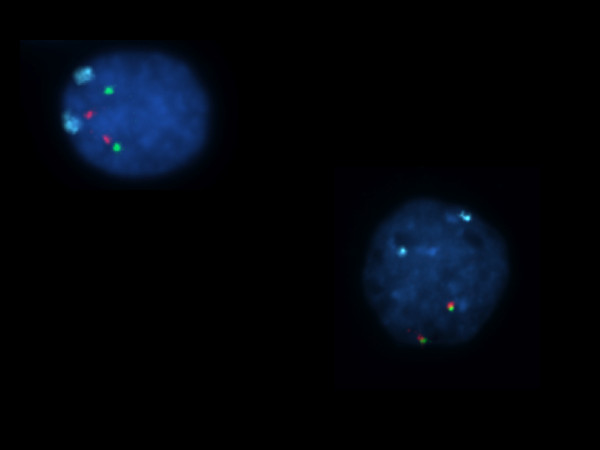
**Two fetal testicular cells showing two dual chromosome 21-specific signals (red and green) and two chromosome 18 control probe signals (ice blue), therefore recorded as being normal disomy 21**.

Using FISH technology with two chromosome 21-specific probes and applying stringent criteria for establishing chromosome 21 copy numbers in the relevant fetal testicular cell nuclei, we could not identify a single T21 cell nucleus in any of these four apparently normal male fetuses in a total cell population of nearly 12.000 (Table 1, Figure 1). We conclude that there is a highly statistically significant sex difference in T21 germ line mosaicism with a much higher incidence in fetal ovaries than testes (p < 0.001). In a previous report we documented an average of 0.54% (range 0.20 - 0.88; SD 0.23) of T21 cell nuclei in fetal ovaries from eight female fetuses [28].

**Table 1:** Results from fluorescent in situ hybridisation (FISH) in fetal testis using two chromosome 21-specific probes (red and green)

		No of signals green/red							
			
Case No/Id	Gest. Age (wks)	2gr/2r	3gr/3r	1gr/1r	2gr/1r	1gr/2r	2gr/3r	3gr/2r	Total no of scored cells
8787	18	3927	-	35*	1	6	2	-	3971
8795	17	2510	-	5	-	2	-	-	2517
5A	14	3294	1#	11**	3	6	-	1	3316
6A	19	2010	-	3	1	22	-	2	2038

Total		11741	1	54	5	36	2	3	11842

*One of these nuclei contained only one chromosome 18 signal and was interpreted as having either monosomy 21 together with monosomy 18 or being haploid; the remaining showing two chromosome 18 signals were recorded as either false negative monosomy 21 (due to somatic pairing) or true monosomy 21 [91,92].

**These nuclei contained two chromosome 18 signals and were also recorded as false negative monosomy 21 (due to somatic pairing) or true monosomy 21[91,92].

#This nucleus had 3 × 18 signals and was recorded as being triploid.

The overriding aim of the study has been to investigate the underlying reasons for the intriguing and largely unexplored aspect of the parental origin of T21 DS, i.e. why the extra free chromosome 21 in T21 DS originates from the mother in more than 90% of cases and from the father in less than 10% [7-11]. It would now appear that before puberty human males hardly harbour any T21 precursor cells able to generate mature 21-disomic sperm cells. Nevertheless, bearing in mind the rare paternal origin, an incidence below one fetal T21 testicular cell per six or seven thousand could still be relevant in this respect.

It is also essential to note that there are a number of Case Reports in the literature, documenting paternal inheritance with either testicular T21 mosaicism identified *per se *or inferred from T21 mosaicism found in somatic tissues, most commonly blood lymphocytes. In addition, there are a number of reports demonstrating a raised incidence of disomy T21 sperm in comparison to controls in fathers of T21 cases. The characteristics of these outstanding cases are summarised in table 2 [30-48]. It has been generally assumed that T21 DS men are infertile, but these reports suggest that at least in cases of T21 mosaicism fertility may be restored. There are also reports of two cases of apparently non-mosaic DS men, who have fathered children [49,50]. T21 DS females, on the other hand, show impaired fertility and premature menopause, but there are many more reports of offspring to apparently non-mosaic DS mothers than DS fathers [51,52].

**Table 2:** Previous studies indicating paternal T21 germ line mosaicism

No. of DS pregnancies	Percentage T21 Cells (%) Paternal Tissue Sample				Proportion of T21 mosaic fathers (%)	Reference
		
	Blood		Skin	Testis/Sperm	Parental dermatoglyphics	
					1	Penrose 1965 [40]

2	*					Massimo et al. 1967 [36]

1	23,3					Walker and Ising 1969 [48]

1 (Family 1)	0		7,5	4 (testicular fibroblasts) 14,3 (spermatocytes) 23 (spermatogonia)		Hsu et al. 1971 [34]
	
1 (Family 2)	6					
	
1 (Family 3)	4,6		4			

					8	Priest et al. 1973 [41]

1	6,7					Mehes et al. 1973 [37]

1 (Case 9)	6,7					Richards 1974 [42]

1	6					Papp et al. 1974 [39]

1 (Familie T)	11					Domány and Métneki 1976 [32]
	
1 (Familie K)	15					

					2	Schmidt et al. 1981 [45]

1	3-5					Rodewald et al. 1981 [43]

1 (Family 8)	1					Uchida et al. 1985 [47]
	
1 (Family 9)	1					

1 (Family 10)	1					
	
3 (Family A)	0		22			Sachs et al. 1990 [44]

2 (Family RDS-02)	2					Pangalos et al. 1992 [38]

1	2					Casati et al. 1992 [31]

1 (DP-4)				0,75 (sperm)		Blanco et al. 1998 [30]
	
1 (DP-5)				0,78 (sperm)		

1 (P19)	1,5					Frias et al. 2002 [33]
	
2 (P24)	1,3					
	
2 (P25)	1,5					

4/13 embryos	0			6,6 (sperm)		Somprasit et al. 2005 [46]

1 (Family A)	*					Kovaleva et al. 2007 [35]
	
1 (Family V)	1,4					
	
1 (Family S)	6,7					

* The proportion of T21 cells was not reported

The data we have here presented raise a number of additional interesting questions, including in particular:

(1) How does the sex difference in fetal germ line T21 mosaicism come about?

(2) Is there a correlation with somatic T21 mosaicism?

(2) What is the reason for the disomy 21 in sperm from normal males?

### How does the sex difference in germ line T21 mosaicism come about?

As judged by investigation of the chromosome constitution in individual cells of embryos at the 8 cell stage (by FISH or array-CGH) a large proportion of such embryos are mosaics including a cell line with an aberrant chromosome number [28,53,54]. These embryos have been obtained by donation from patients undergoing IVF treatment, but it is generally thought that the same propensity to embryonic aneuploidy mosaicism is equally common in embryos conceived naturally.

The reasons for this early segregation failure as regards a single or a few chromosomes are not known. Neither is it clear what the impact may be of this phenomenon at later cell divisions during the window from the first to the fifth week of fetal life, preceding the differentiation of the gonads into ovaries and testes. It seems likely, however, that some 'self-correction' can take place [55,56]. Indeed, any occurrence and survival of T21 stem cells at this early stage should not, conceivably, differ in either sex.

The germ cell precursors, the primordial germ cells, are differentiated among the endodermal cells of the yolk sac already at around four weeks of fetal life. They then migrate to the gonadal ridge during the following week. We have previously proposed [27] that the T21 fetal ovarian mosaicism detected at 14-22 weeks has been caused by oogonial mal-segregation starting at around five weeks gestational age, i.e. when the migrating germ cells have reached their final destination in the mesenchyme of the urogenital ridge [57-60]. Tentatively we may suggest that one likely reason for the sexual dimorphism in this respect with a much lower incidence of T21 mosaicism in fetal testes, if any, is a more stringent control of the corresponding cell divisions in fetal testes than in ovaries. In a broader sense, the very same selective mechanism has been invoked to account for the higher proportion of DS mothers than fathers with the typical dermatoglyphic DS patterns [43].

### Is there a correlation with somatic T21 mosaicism?

It would be of further interest to ascertain the relation between this newly discovered sex difference in gonadal development and that affecting the soma. If we are right in our assumption that the common fetal ovarian T21 mosaicism identified in our previous study [28] is exclusively due to a less stringent control of chromosome segregation during fetal oogonial development than during the corresponding cell divisions (the gonocytes, the intermediate cells and the pre-spermatogonia) in fetal testes, then we would not expect a correlation with T21 mosaicism in somatic cells. Yet again, further investigations will be required, analysing a number of different types of fetal somatic tissues as well as germ cells to answer this outstanding question. One other aspect of this question concerns the possibility that T21 mosaicism might be induced by environmental agents including that seen in miscarriages [29,61-66].

It is further well known that some parents of T21 DS children are themselves T21 mosaics in both somatic cell populations and in the germ line. Interestingly, there are in this category of DS parents a larger number of women than men [47,67-69]. In addition there is a sex difference also as regards uniparental disomy (UPD) caused by so-called rescue in an original T21 zygote, this type of mosaicism again being more common in females than males [70-72]. The question then arises if T21 mosaicism involving both the germ line and the soma might more often be due to rescue during the subsequent cell divisions in an original T21 zygote rather than mal-segregation in an embryo/fetus that was originally normal euploid, containing two chromosomes 21 [73]. If the latter were to apply (and in the absence of somatic crossing-over) all ensuing cases should be isodisomic for two of the three chromosomes 21, making up this somatically acquired aneuploidy. There are a number of studies showing the typical DS dermatoglyphic pattern in parents and sibs substantiating the notion that the trisomic cell line was indeed passed down from a mother to an affected proband [43,45,74-76]. This type of mechanism would also agree with the observation of Katz-Jaffe et al [77] that it is only the derivatives of initially T21 zygotes, which contribute to the T21 amniocyte population recovered as such.

### What is the reason for the disomy 21 in sperm from normal males?

Finally, there are by comparison a large number of publications (to date totalling at least 34), recording rate of disomy 21 in sperm from apparently normal controls. Results vary quite substantially in estimates of disomy 21 in individual sperm samples from 0.00 - 0.44% [78-84]. Some of these discrepancies might be caused by technological problems in FISH analysis. In a previous investigation (on amniocytes) the apparent false positive signals using a single chromosome 21 probe amounted to around 1% of cells analysed [85]. The implication of this consideration is that further studies on spermatozoa obtained from normal control men using two chromosome 21-specific probes will be required before we may be certain what the true incidence is of sperm disomy 21 in the normal population.

At the moment we can only surmise that any such disomy 21 may occur by mal-segregation/nondisjunction of chromosome 21 at pre-meiotic spermatogonial divisions and/or the later meiotic Anaphase I and Anaphase II stages of spermatogenesis in adult men. To our knowledge there are no relevant previous studies investigating chromosome segregation in testicular biopsy samples from normal adult men. In two previous small studies evaluating chromosome number in secondary spermatocytes at the Metaphase II stage, there was no indication of an extra chromosome 21 by analysis of 266 cells at this meiotic stage in testicular biopsy samples from adult men [86,87]. Interestingly, however, a correlation has been found between incidence of disomy 21 in spermatozoa and T21 in blood lymphocytes in both normal fertile controls and men suffering from subfertility [78,80,81].

## Conclusion

In this paper we have documented copy number counts of chromosome 21 by fluorescence in situ hybridisation (FISH) on fetal testes obtained from four apparently normal male fetuses following termination of pregnancy for a non-medical/social reason. Applying stringent criteria for identification of T21 in germ cell nuclei we could not detect a single T21 cell in a population of at least 2000 per case. We conclude that there is a substantial sex difference in incidence of fetal germ line T21 mosaicism where most female fetuses may be ovarian T21 mosaics, while males in this study do not show any detectable degree of fetal testicular T21 mosaicism. We propose that this sex difference in germ line T21 mosaicism may explain the much lower paternal origin of T21 DS than maternal. The mechanisms underlying the rare DS cases where the extra chromosome 21 does originate from the father, remains unknown and further studies in this respect are required.

As we have stressed in our previous publication on this issue [27,28] further large-scale studies will be required to find out if our model on germ line mosaicism leading to secondary meiotic non-disjunction constitutes the major source of aneuploid conceptions in the human population, or if other mechanisms might also contribute to this effect.

## Materials and methods

All procedures were performed with informed consent and ethical approval from the local ethical committee. Fetal testicular cells were obtained from four fetuses at gestational age 14-19 weeks, following termination of pregnancy for social reasons with all the fetuses having a normal phenotypic appearance. Testes were removed within a few hours post-mortem and placed in L-15 (Leibovitz) medium (Life Technologies) with 0.3% bovine serum albumin (Sigma). Pieces of testes were frozen at -80°C. Parts of the tissue samples were thawed to prepare direct imprints from the cut surface of the fetal ovary [88] and the remaining material processed by micro-spreading [89].

Microscopy slides for FISH analysis were fixed in methanol: acetic acid (3:1 v/v) then washed in 2× standard saline citrate (SSC) and treated with pepsin (0.1 mg/ml) in 0.01 M HCl for 8 min at 37°C. After additional washing in phosphate-buffered saline (PBS), paraformaldehyde (1%) fixation and dehydration through series of alcohol the slides were left to air-dry at room temperature. Hybridisation was performed according to the manufacturers' instructions with two DNA probes positioned near the end of the long arm of chromosome 21 and labelled in SpectrumOrange and SpectrumGreen respectively (Cat No: 32-190002, Abbot Molecular Inc, USA and Cytocell, Cat No. LPT21QG/R, Cytocell Technologies Ltd. UK). A chromosome 18 centromeric probe labelled in SpectrumAqua was added to be able to differentiate between trisomy and triploidy (Cat No: 32-131018, CEP 18 (D18Z1) SpectrumAqua Probe). The DNA probes were mixed and added to the slides followed by denaturation, hybridisation and post-hybridisation washing. After dehydration slides were mounted in glycerol containing 2.3% DABCO (1, 4-diazabicyclo-(2, 2, 2) octane) as antifade and DAPI (4, 6,-diamino-2-phenyl-indole) 0.5 mg/ml for nuclear counterstaining.

Fluorescent signals were analyzed using a Zeiss Axioskop 2 microscope equipped with a cooled CCD camera (CoolSnap; Photometrics Ltd, USA) controlled by a Power Macintosh computer. Grey scale images were captured, pseudocolored and merged using the SmartCapture 2 software (Digital Scientific Ltd, UK).

In scoring chromosome 21 copy number we focussed attention in particular on the testicular germ cells, i.e. the gonocytes, the intermediate cells and the pre-spermatogonia, identified by their specific morphology [90]. The images of two cell nuclei, showing two dual chromosome 21-specific signals (red and green) and two chromosome 18 control probe signals (ice blue) are illustrated in Fig 1.

## Competing interests

The authors declare that they have no competing interests.

## Authors' contributions

MH designed the study, supervised the practical work and wrote the initial draft of the paper; EI and SP performed the FISH analysis; MW and NP obtained local ethical approval; EI, MW, NP and AMJ obtained the samples. All the authors contributed to and have approved the final version of the manuscript.
